# Large intramuscular hematoma due to acquired Factor VIII inhibitors in post Polycythemia Vera-Myelofibrosis

**DOI:** 10.46989/001c.141157

**Published:** 2025-07-07

**Authors:** Raghuveer S. Prabhu, Sarath R. V.S., Rahmathullah S. N.

**Affiliations:** 1 Department of Hematology, IQRAA International Hospital and Research Center, Wayanad Road, Malaparamba, Kozhikode PIN– 673 002, Kerala, India; 2 Department of Radiology, IQRAA International Hospital and Research Center, Wayanad Road, Malaparamba, Kozhikode PIN– 673 002, Kerala, India

**Keywords:** Myeloproliferative Disorders, Hematoma, Hemophilia A, Blood Coagulation Disorders, Factor 8 deficiency acquired

## Abstract

A 59-year-old man with Janus kinase-2 (JAK2) V617F mutation-positive polycythemia vera, evolving to myelofibrosis presented with a right thigh hematoma. Further evaluation showed prolonged activated partial thromboplastin time (aPTT), which was partially corrected after mixing with pooled normal plasma (PNP) and, low factor VIII (F VIII) levels. He was diagnosed to have acquired F VIII inhibitors, and treated with prednisolone for inhibitor eradication. After four weeks of treatment, his aPTT normalized, F VIII rose to 86% and the hematoma was resolved. The case report is followed by a discussion on the topic, revisiting the handful of cases published so far, and the possible mechanisms leading to inhibitor formation in MPN. Further studies are required to elucidate the pathophysiology and the incidence of F VIII inhibitor development in myeloproliferative neoplasms.

## Introduction

Acquired inhibitors to Factor VIII (F VIII) is a rare disease, with an incidence of 1–4 per million/year.^1^ Fifty percent of the cases are idiopathic, and often occur in the elderly. Post partum state, autoimmune disorders, malignancies, infections and drugs constitute the rest of the cases.^1^ Myeloproliferative neoplasms (MPN) are characterized by abnormal proliferation of one or more terminal myeloid cell lines in the peripheral blood. Philadelphia chromosome negative MPN occurs with a frequency of 22–74 cases per million per year.^2^ MPN is not widely known to lead to acquired F VIII inhibitors. Only a handful of cases have been reported in the published literature so far. We describe a 59year old patient with polycythemia vera evolving to myelofibrosis (PV-MF), who presented with large intramuscular hematoma and, in whom subsequent evaluation led to a diagnosis of acquired hemophilia A.

## Case Report

A 59-year old man presented with one month history of painful swelling over the right thigh. Eight months before, he had been evaluated for high blood counts – hemoglobin, white blood cell (WBC) and platelet counts were elevated. A bone marrow biopsy showed panmyelosis with grade-2 reticulin fibrosis. Janus kinase-2 (JAK2) V617F mutation was detected in his peripheral blood. He had been diagnosed with PV-MF. He reexperienced more than usual bleeding after bone marrow biopsy, which settled with local measures. He did not report any history of excess bleeding-related events prior to the diagnosis of PV-MF. Since the diagnosis of PV-MF, he was requiring regular therapeutic phlebotomies and was on aspirin 75 mg per day. He had been started on hydroxyurea one month prior to the current presentation.

**Figure 1.**
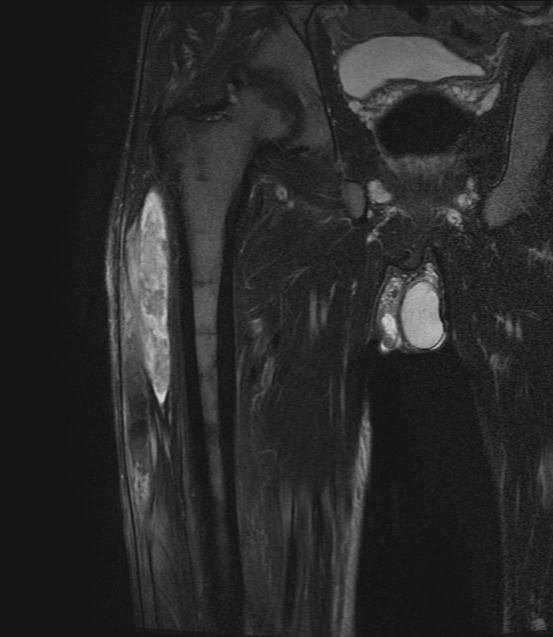
Coronal T2-weighted fat-saturated sequence shows the cranio-caudal extension of the hematoma in the thigh.

On presentation, he had a tender indurated swelling of 20 cm length and 5 cm width, along the lateral aspect of the right thigh. There was local rise of temperature over the site. Systemic examination revealed palpable splenomegaly 5 cm below the left costal margin. He also had left-side inguinal hernia. Blood examination showed hemoglobin (Hb) of 15.3 g/dL, hematocrit 47 %, an elevated WBC count of 14500 cells/µL, and high platelet count 716000/ µL. Lactate dehydrogenase (LDH) was elevated, at 374 U/L. Magnetic Resonance Imaging (MRI) scan of the right thigh (Figure 1) revealed elongated altered intensity signal lesion (hyperintense in T1- weighted image, T2-weighted fat-saturated image) in the lateral aspect of right thigh, measuring 29.5 x 7.2 x 2.4 cm, suggestive of a large hematoma. There was no muscle necrosis nor compression of neurovascular structures.

A coagulation work-up (Table 1) showed prolonged activated partial thromboplastin time (aPTT) of 48 sec (normal range 26.7 – 32.9), with only partial correction to 42.4 sec after mixing with pooled normal plasma (PNP) and 2-h incubation of the mix at 37°C. There was no correction after mixing with factor VIII deficient plasma, indicating F VIII deficiency. Factor VIII (F VIII) levels were low – 25%. Inhibitor titre could not be established due to resource constraints. Prothrombin Time (PT) and Thrombin Time (TT) were normal. Factor IX (91%) and von Willebrand Factor (vWF) antigen (49.6%) were normal.

**Table 1.** Coagulation parameters

	**Test**	**Test Value**	**Normal Range**
1	Blood group	‘O’ positive	
2	Bleeding Time	2.30 minutes	2.0-6.0 minutes
3	Prothrombin Time	14.5 Seconds	12.5-15.5 Seconds
4	APTT	42.4 Seconds	26.7-32.9 Seconds
5	Mixing Study APTT (½ test + ½ PNP) incubated for 2 hours at 37°C	37.0 Seconds	
6	Mixing Study APTT (½ test + ½ Factor VIII deficient plasma)	48.0 Seconds	
7	Thrombin Time	17.3 Seconds	<20 Seconds
8	Factor XIII screen (urea clot solubility test)	Normal	Normal
9	Platelet Count	8.04 L	1.5-4.1 L
10	Direct Smear	Increased platelets seen in singles and large clumps	
11	Factor VIII assay	25.0 %	50-150 %
12	Factor IX assay	91.0 %	50-150 %
13	vWF:Ag assay	49.6 %*	60-200 %

APTT – activated partial thromboplastin time, PNP – pooled normal plasma, vWF:Ag – von Willebrand Factor: Antigen *‘O’ blood group persons have vWF:Ag 25 % below the reference range. So this value is considered normal

Acquired Von Willebrand syndrome (aVWS), which is a more common cause of bleeding among MPN patients, was unlikely, as VWF antigen level and bleeding time were normal. Also, the clinical presentation with intramuscular hematoma was not typical for aVWS, which usually presents with mucosal or skin bleeds. VWF functional assays and multimer analysis would have conclusively ruled out aVWS but could not be done as the tests were not available at our center. As Factor IX levels were normal, acquired Factor IX deficiency was ruled out. In view of prolonged aPTT, which was not corrected with 1:1 mix of F VIII deficient plasma incubated, and only partially corrected with 1:1 mix PNP incubated for 2 hours at 37°C, a diagnosis of acquired Hemophilia A related to MPN, PV-MF was made. He was started on 1 mg/kg/day oral prednisolone for inhibitor eradication. With this therapy, his aPTT normalized and the factor VIII levels increased to 86% at 4 weeks. His thigh hematoma gradually resolved. As the clinical signs of hematoma completely disappeared and due to resource constraints, a repeat imaging was not done to reassess the hematoma. Two months later, the patient successfully underwent surgery for inguinal hernia repair without any factor concentrate/blood product support.

## Discussion

Ever since the first documented report of paradoxical bleeding in MPN in 1895,^3^ bleeding has been observed regularly in these patients who are also prone to thrombotic events. It was not until 1999 that the underlying mechanism for the bleeding manifestations in MPN was elucidated. A report by Michiels.^4^ indicated that aVWS due to increased platelet count led to bleeding manifestations in these patients. In these cases, bleeding occurs due to the loss of intermediate and large multimers of vWF associated with extremely high platelet counts above 100,00,00/ µL.

Acquired F VIII inhibitors as another mechanism for bleeding in patients with MPN was first reported in 2011, by Aledort and Kremyanskaya.^5^ They described a 74-year-old individual with JAK2+ essential thrombocythemia (ET) who presented with a left arm hematoma followed by a right forearm hematoma with compartment syndrome. She had a prolonged aPTT of 66.5 sec and normal PT; mixing study showed the presence of circulating inhibitor and a F VIII level of 4%. In contrast, our patient developed a massive hematoma even with mild reduction in F VIII (25%). We propose that along with acquired F VIII inhibitor, the antiplatelet effects of aspirin might have contributed to his hematoma formation.

Acquired inhibitors against FVIII, also termed acquired hemophilia A, is a rare occurrence, with an incidence of approximately 1 to 4 per million/year.^1^ It is characterized by soft tissue hemorrhage and mucosal bleeds into the gastrointestinal/urologic tracts, and is associated with increased morbidity and mortality.^6^ This is in contrast with congenital F VIII deficiency, where hemarthroses are the major manifestation. Acquired F VIII inhibitors is an autoimmune condition, which is more common in the elderly and, in up to 50% of cases, is idiopathic. In the remaining cases it is related to post-partum state, autoimmune disorders, solid or hematologic malignancies, infections and drugs. Hematological malignancies commonly associated with this condition are chronic lymphocytic leukemia (CLL), non-Hodgkin lymphoma (NHL), multiple myeloma and Waldenström macroglobulinemia.^7^ Fludarabine, a drug used in the treatment of CLL and NHL, can also rarely lead to acquired hemophilia A.^8^

MPN, leading to acquired F VIII inhibitors is a rare occurrence. After the initial report in 2011, only a handful of cases have been published. Mori et al. reported acquired hemophilia A in a 69-year-old lady with ET, who presented with giant ecchymoses, prolonged aPTT, F VIII < 1% and high Bethesda titre, and who rapidly succumbed to her illness.^9^ Wrobel et al described a 66-year-old man with myelofibrosis evolving to acute myeloid leukemia who was diagnosed as acquired F VIII inhibitors, following evaluation for post operative bleeding.^10^ This was controlled with recombinant factor VII, and the F VIII inhibitors were eradicated with methyl prednisolone and rituximab. Vener et al. reported a 64-year-old man with mutated JAK2 exon 12 PV-MF, developing severe retroperitoneal hemorrhage and psoas muscle hematoma, following the formation of acquired F VIII inhibitors.^11^ Gulbicka et al. described a 76-year-old man with JAK2 positive PV-MF who developed massive chest wall and abdominopelvic muscle hematomas, had aPTT of 60 secs, low F VIII levels, inhibitor titre of 0.6 Bethesda units, and was diagnosed to have acquired F VIII inhibitors.^12^ The presentation in our patient was identical to the latter two cases, in that he had PV-MF and developed muscle hematoma when F VIII inhibitors were formed. Table 2 shows the comparison between the current and the previously reported cases of acquired F VIII inhibitors among MPN patients.

**Table 2.** Comparison of cases of acquired hemophilia A among MPN patients

Reference number	Diagnosis	Age (years)	Mutation status	Site of bleeding	Factor VIII level (%)	Inhibitor titre	Therapy for inhibitor eradication	Outcome
5	ET	74	JAK2 mutated	Forearm muscles	4	5.8 BU	Prednisolone	Inhibitor eradicated
9	ET	69	Not mentioned	Subcutaneous, intracerebral	< 1%	17 BU	Nil	Died
10	PMF transformed to acute leukemia	66	Not mentioned	Post-operative, intra-abdominal hematoma	< 1%	17.3 BU	IV methyl prednisolone, rituximab	Partial response
11	PV-MF	57	JAK2 exon 12 mutated	Retroperitoneal hemorrhage, posas muscle, subcutaneous, nasal mucosa	< 1%	90 BU	Prednisolone	Inhibitor eradicated
12	PV-MF	72	JAK2 mutated	Chest wall and abdominopelvic cavity	Decreased	0.6 BU	Prednisolone, Cyclophosphamide	Inhibitor eradicated
Current case	PV-MF	59	JAK2 V617F mutated	Thigh muscle	25 %	Not done	Prednisolone	Inhibitor eradicated

BU- Bethesda units, ET- Essential thrombocytosis, JAK2- Janus Kinase2, PMF – Primary Myelofibrosis, PV-MF- Polycythemia vera transformed to myelofibrosis

Patients with MPN are prone to both thrombosis and bleeding. After PV, PV-MF has the highest risk of thrombosis among the MPN.^13^ This is thought to be due to platelet activation and subsequent platelet-leukocyte adherence and endothelial cell activation, whereas the bleeding events in MPN are usually related to the high platelet counts, leading to clearance of high molecular weight VWF multimers, i.e., due to aVWS. As mentioned above, acquired F VIII inhibitors were only recently recognized as a cause of bleeding among MPN patients. Hence, data regarding such cases are scarce.

The present case is only the sixth instance of acquired F VIII inhibitors in patients with MPN. Of the previous five cases, three had myelofibrosis, of which two were PV-MF as in our patient. The median interval for the development of myelofibrosis in a case of PV is 13 years (range 2.4 to 29.6) from the diagnosis of PV.^14^ We propose that the development of acquired inhibitors to F VIII is a time-dependent phenomenon in patients with MPN. The constitutive activation of the JAK-STAT3 pathway occurs in MPN. Autoimmunity can be a result of the constant activation of the JAK-STAT3 signaling pathway, leading to a reduction in immunosuppressive Tregs and an increase in autoimmune Th17 cells.^15^ Polymorphisms and mutations in the JAK-STAT pathways have been linked to auto-immune conditions and immune-mediated cancers, as shown in multiple genome-wide analysis.^16,17^ The rs324011 polymorphism in STAT6 results in early immune dysregulation with depressed Treg function and increased Th1 response.^18^ There are links between STAT3 gain-of-function mutations and early-onset autoimmunity.^19^ IgG anti-endothelial cell antibodies (AECA) are increased in certain cases of MPN.^14^ Factor VIII is synthesized from the endothelial cells. We propose that injury to these cells by the AECA may result in exposing certain epitopes in the Factor VIII molecule, which can then lead to development of acquired inhibitors against F VIII in the setting of activated JAK-STAT3 signaling. The inflammation caused by AECA in the setting of constitutive JAK-STAT signaling creates a milieu for formation of F VIII inhibitors. Understanding the above pathophysiology behind the formation of F VIII inhibitors in MPN, throws open a question regarding the potential for JAK inhibitors, like ruxolitinib, pacritinib and momelotinib, in modulating the immune response and, thus, preventing the formation of inhibitors. More focused research is essential in the field of acquired F VIII inhibitors in MPN to better understand the pathophysiology and to develop preventive/pre-emptive therapeutic strategies. Prospective studies are required to accurately determine the incidence of acquired F VIII inhibitors in MPN, and to determine whether early initiation of JAK inhibitors can reduce the incidence of inhibitor formation.

### Authors’ Contribution

R.S.P. and R.S.N. conceptualized the study, R.S.N. and S.R.V.S. collected the data, R.S.P. and S.R.V.S. analysed the data, R.S.P. and S.R.V.S. wrote the draft manuscript, R.S.N. and S.R.V.S. edited the manuscript, R.S.P. revised the manuscript. All authors approved the final manuscript.

### Competing Interest – COPE

All the authors do not have any financial or nonfinancial interests to disclose. Authors did not receive any funding for the study.

### Informed Consent Statement

All authors and institutions have confirmed this manuscript for publication.

## Data Availability

All are available upon reasonable request.
